# Evaluating the Link Between Postoperative Timing of Rifampicin Introduction and the Clinical and Microbiological Outcomes of Orthopedic Staphylococcal Implant Infections

**DOI:** 10.3390/antibiotics14101043

**Published:** 2025-10-17

**Authors:** Valeria Dessert, Steven M. Maurer, Marc S. Maurer, David Albrecht, Mazda Farshad, İlker Uçkay

**Affiliations:** 1 Internal Medicine, Balgrist University Hospital, University of Zurich, 8008 Zurich, Switzerland; valeria.dessert@balgrist.ch; 2 Department of Orthopedic Surgery, Balgrist University Hospital, University of Zurich, 8008 Zurich, Switzerland; steven_maurer@hotmail.com (S.M.M.); mazda.farshad@balgrist.ch (M.F.); 3 Infectiology, Unit for Clinical and Applied Research, Balgrist University Hospital, University of Zurich, 8008 Zurich, Switzerland

**Keywords:** implant infections, rifampicin, delay of introduction, rifampicin resistance, clinical outcomes, orthopedic surgery

## Abstract

Background/Objectives: In staphylococcal implant infections, there is often discussion about the optimal postoperative timing of the introduction of rifampicin in the postoperative period with open wounds. Methods: We reviewed all adult patients with residual staphylococcal implant infections between January 2014 and May 2024. We analyzed the delay to rifampicin use in relation to therapeutic failures, infection recurrences, and development of ultimate rifampicin resistance. Results: Among 103 independent infection episodes, 47 (46%) contained the pathogen *S. aureus*, and the remainder were different coagulase-negative staphylococci. The median number of surgical interventions was one, and the median duration of postsurgical systemic antibiotic treatment was 84 days (interquartile range (IQR), 42–84 d). The median daily dose of oral rifampicin was 900 mg, and the median delay in its introduction was 5 days (IQR, 3–8 d). Overall, 18% of patients experienced an adverse event related to rifampicin (mostly gastrointestinal), requiring treatment to be stopped. The incidences of clinical failures and of microbiologically identical recurrences were 27% and 10%, respectively. The risk of rifampin resistance among any new staphylococcal infection or colonization during a median follow-up of 1.9 years was 1%. In the multivariate Cox regression analysis, the delay in rifampicin administration, its dose, or its duration failed to alter outcomes. Conclusions: In our retrospective cohort of staphylococcal orthopedic implant infections, the timing of rifampicin introduction failed to alter clinical and microbiological outcomes.

## 1. Introduction

For decades, surgeons and infectious diseases physicians have added rifampin/rifampicin (RIFA) to the postoperative antibiotic treatment of staphylococcal implant infections if the material is retained or subjected to a one-stage exchange [1]. The biofilm is a matrix found on non-living foreign bodies such as implants, and it is composed of proteins, sugars, debris, and bacteria, representing a major therapeutic problem in staphylococcal implant infections [2]. Antibiotics and leukocytes penetrate poorly into bone, and the penetration into the biofilm of non-irrigated foreign bodies is even more difficult [2]. Moreover, in this matrix, the majority of bacteria live in a dormant state, with reduced or minimal metabolic activity. This lack of metabolism is important because most antibiotics only kill dividing bacteria, or those with an enhanced metabolic activity, on the cell walls. In contrast, there are also antibiotic agents acting inside a “dormant” cell such as RIFA. Most experts consider RIFA the best antimicrobial agent that could penetrate biofilms and kill these embedded bacteria. Therefore, RIFA is a very precious molecule against which staphylococci must never develop resistance [1,3]. RIFA resistance can occur very rapidly (within a few days) in cases of RIFA monotherapy [4,5] or a high inoculum (i.e., bacteremia, lack of adequate debridement, or entry of new staphylococci via open wounds). Therefore, most experts advocate combining RIFA with another agent throughout the entire therapeutic course [3], which contradicts the common practice of awaiting wound closure. 

In daily clinical practice, combined therapy with RIFA does not occur on Day 0 for many reasons, such as a high inoculum of pathogens that cannot be removed, e.g., in cases of bacteremia; strong nausea in the surgical aftermath; and the presence of orthopedic wounds that are still discharging. Many clinicians believe that wound healing problems are caused by a high inoculum of underlying bacteria or the acquisition of new (resistant) strains from the surface for which current therapeutic agents, e.g., RIFA, would act as selectors of new (and more resistant) strains [3,4,5,6]. Clinicians must decide between a theoretical risk of future RIFA resistance and the deprivation of the best regimen in the critical early phase of biofilm formation [6]. We believe that this is a very rare risk, which motivated us to investigate this widespread practice of awaiting wound closure. The purpose of this study is to investigate whether the timing of RIFA introduction influences clinical outcomes, recurrence, or resistance development. We do not investigate the efficacy of combining RIFA with other agents to treat staphylococcal implant infections as a large volume of literature is available on this topic [1].

## 2. Results

### 2.1. Patients and Staphylococcal Implant Infections

**Figure 1:**
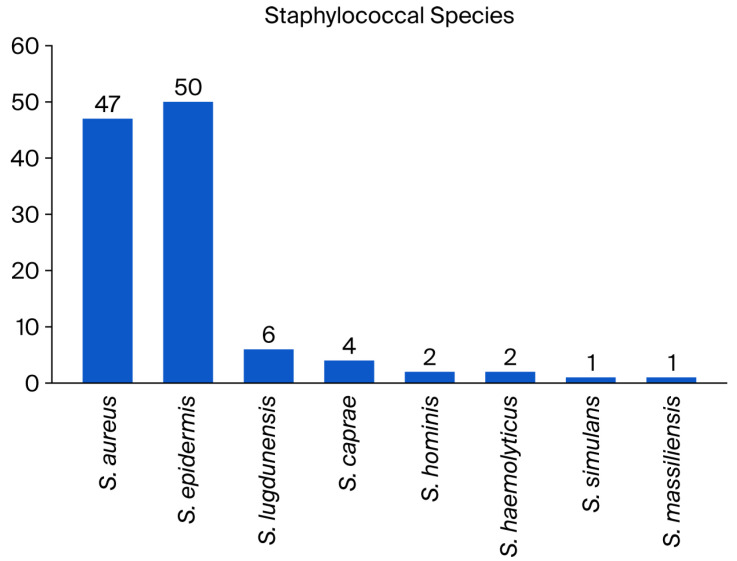
Distribution of causative pathogens according to their species.

Among 242 independent infection episodes due to various staphylococci and orthopedic implants, 103 fulfilled our study criteria (43 % female patients). Infection sites included the knee (*n* = 31; 30%), hip (24; 22%), spine (33; 32%), foot (7; 7%), shoulder (5; 5%), and hand (1; 1%). Implant types comprised total joint arthroplasties (*n* = 54; 52%), plates (11; 11%), screws (2%), spondylosis and cages (33; 32%), cerclages (1%), and pins (1%). We noted 26 distinct staphylococcal constellations, with *Staphylococcus aureus* predominating in 47 cases (46%), including three healthcare-associated and one community-acquired MRSA (methicillin-resistant *S. aureus*). The group of coagulase-negative staphylococci comprised 56 cases (54%; Figure 1). The pathogens were methicillin-resistant in half of the episodes (49%) and polymicrobial in 19 (18%). The median active follow-up (surgical control visits) lasted 1.9 years (interquartile range (IQR), 1.0–2.7 y), and the median passive follow-up was 2.9 years (IQR, 2.3–3.9 y). We closed the database on 10 July 2025. According to the files on infectious diseases, the included patients have not been previously exposed to RIFA therapy, such as for latent tuberculosis.

### 2.2. Surgical and Medical Therapy with Combined RIFA Use

The median number of surgical debridements per episode was one (IQR, 1–2 surgeries). We used 22 different initial empirical systemic antibiotic regimens, of which the three most frequent agents were co-amoxiclav (*n* = 49), vancomycin (*n* = 21), and cefazolin (*n* = 5). We did not use local antibiotic-loaded cements or intraosseous local antibiotics for retained implants. The total median duration of the postoperative antibiotic treatment was 84 days (IQR, 42–84 d), while that of oral RIFA use was 70 days (IQR, 39–80 d), resulting in a median RIFA-to-beta-lactam ratio of 0.92 for the antibiotic course (IQR, ratio 0.82–0.96). The median delay until postoperative RIFA introduction was five days (IQR, 3–8 d). We prescribed RIFA in a median daily dose of 900 mg (450 mg bid), and rifampicin was used as the agent of RIFA in all cases. Switzerland almost never uses rifampin or rifabutin. Overall, 19 patients (19/103; 18%) experienced a significant adverse event (AE) attributed by clinicians to RIFA use, including appetite loss (*n* = 8; 8%), emesis (5%), protracted nausea (3%), oral mycosis (2%), and probable skin rash (2%). The occurrence of RIFA-related AEs significantly reduced the duration of the RIFA part of therapy (median of 73 days without vs. 41 days with adverse events; Wilcoxon rank sum test; *p* = 0.01). Of note, the RIFA dose did not increase the risk for AEs (median 900 mg vs. 900 mg; *p* = 0.55). 

### 2.3. Outcomes

We observed 27 episodes of “clinical failures” after the first round of therapy: new infection (*n* = 13), recurrent infection (7), persistent infection (3), invalidating back pain (2), material failure (1), bone instability (1), and hematoma requiring re-intervention (*n* = 1), while one episode might reveal several reasons for failure. During and after treatment, the surgeons performed a revision in 42 cases (40%) for reasons other than clinical failures, including implant removal for mechanical reasons (*n* = 3), luxation of a temporary spacer (1), scar revisions (3), arthrofibrosis (1), second looks (15), and mechanical correction (*n* = 4). The case mix was broad. Table 1 reveals the key results of our adjustment with Cox regression, with an emphasis on RIFA-related variables. 

The incidence of “microbiological recurrences” with the same *Staphylococcus* strain was 9.7% (10/103 episodes). A group comparison or a multivariate analysis was not possible due to the small number (*n* = 10) of this variable. During the long study follow-up, six patients died from causes unrelated to orthopedic infections or RIFA use. The median length of hospital stay in our acute surgical wards was 12 days (IQR, 8–17 d).

### 2.4. Rifampicin Resistance

The median delay between the first debridement and the “last *Staphylococcus*” (sampled for any indication) was 322 days (IQR, 42–425 d). The staphylococcal species between the index pathogen and the “last *Staphylococcus*” differed in seven cases (7%). In sixteen episodes (16%), the initial pathogen and the later control belonged to the same species but were different in their susceptibility testing according to at least three antibiotic classes. Among the 103 implant infections, two therapies were started with a RIFA-resistant strain. Both lost their resistance, replacing the initial stain with a RIFA-susceptible strain. 

In only one case of a periprosthetic knee joint infection due to methicillin-resistant *S. epidermidis* (1/103; 1%), the same initial RIFA-susceptible *S. epidermidis* strain became RIFA-resistant “after” 68 days of RIFA treatment. In this case, the initial delay in RIFA introduction was 8 days, which is longer but not significantly different from the other 102 cases (median 5 days). Additionally, RIFA duration lasted 34 days (compared to a median of 84 days among the controls; *p* = 0.19), and its daily oral dose was 450 mg bid (*p* = 1.00). The accompanying agent for 42 days of total therapy was co-amoxiclav.

### 2.5. Literature Review

The first and last authors performed a narrative literature review focusing on the timing of RIFA initiation. We summarize its key findings in Supplementary File S1.

## 3. Discussion

According to our experience, the incidence of a (future) RIFA-resistant strain after long-term combination antibiotic treatment with RIFA was low (1%) regardless of the delay in RIFA introduction, the daily dose, and the total duration of its administration. Our findings are in line with our clinical experience, the sparse literature regarding RIFA resistance [7], and the clinical failure rates of staphylococcal orthopedic infections [8,9,10]. RIFA resistance is usually related to a spontaneous chromosomal mutation in the *rpoB* gene [11,12]. RIFA resistance is more likely linked to the acquisition of specific strains with an individual genetic background, staphylococcal cassettes, and clonality [13,14], which replace former causes. We observed two infected patients starting with a RIFA resistance strain who later switched to susceptible strains and only one case of genuine resistance development. 

The epidemiology of RIFA resistance among staphylococci is complex and heterogenous across geography and time [11]. In Italy and Southern Germany, RIFA resistance has increased to 16% among multidrug-resistant MRSA isolates compared to the rest of Europe (5.7%) [14,15], but not ubiquitously. To cite a positive example, Grünwald et al. investigated 212 patients with prosthetic joint infections undergoing RIFA treatment. Only 0.9% patients after the first stage and 1.4% at the follow-up developed resistance [6], concluding that RIFA administration could be started on the second postoperative day when sufficient concentrations of the accompanying antibiotics can be expected [6]. RIFA resistance might disappear over time [7], for example, when facing competition. In animals infected with equal numbers of RIFA-resistant and RIFA-susceptible bacteria, only RIFA-susceptible strains were recovered after four weeks, indicating that they outcompeted RIFA-resistant isolates [13]. Moreover, select RIFA resistance cases may not persist in initially RIFA-susceptible infections following the discontinuation of RIFA because they simply may not have competitive fitness [7,13]. Unsurprisingly, RIFA-based combination therapy may remain active in implant infections regardless of resistance following the initial selection of RIFA resistance after prior RIFA monotherapy [7].

RIFA combinations are established in modern-day infectiology for staphylococcal biofilm infections. Animal cage models show impressive rapid curves of RIFA combinations, but clinicians cannot reproduce in vitro results [1] and instead observe a high prevalence of RIFA-related AEs [16,17]. The landmark study of Prof. Zimmerli in the early 1990s showed that a combination of ciprofloxacin and RIFA was superior to ciprofloxacin alone for the treatment of staphylococcal implant-related biofilms [18]. This prospective randomized trial was halted only after the inclusion of 33 episodes because of a significant advantage of the combination. The success study could never be repeated, partly because no ID physician would use ciprofloxacin in monotherapy (an anti-Gram-negative agent) for complicated staphylococcal infections today, even if the oral bioavailability and bone penetration of oral ciprofloxacin are good [19]. Subsequent trials regularly failed to show this initial impressive benefit when combining RIFA with ciprofloxacin [20] or other antibiotic agents [20]. Researchers mostly detect a superiority of RIFA combinations in select groups such as MRSA infections [10,21] or in select surgical approaches such as implant retention procedures [22,23,24]. Kruse et al. recently reached the opposite conclusion in their Canadian meta-analysis of clinical studies [25]. They found that the protective effect of RIFA was maintained in studies which included exchange arthroplasty, but not in studies using an implant retention strategy [25]. Several meta-analyses confirmed that RIFA combinations revealed higher cure rates than monotherapies in cohort studies, but not in randomized controlled trials [22,23,26,27], and the certainty and quality of evidence were low [22,23]. The future will be marked by discussions about which patients benefit from RIFA combinations [3]. 

The optimal timing of RIFA initiation remains an unresolved controversy with sparse studies. In a very large multicenter study in the Rhône-Alpes region of France, involving six centers with infected hip and knee total joint arthroplasties, the authors failed to observe a significant difference between success and failure in overall RIFA use, dose, and, importantly, the delay in its introduction [24]. Only the duration of RIFA therapy was associated with failure. Kaplan–Meier estimates of failure were higher in patients receiving less than 14 days of RIFA in comparison with those receiving more than 14 days [24]. We are aware of only one paper advocating that delaying RIFA treatment would be better, albeit this conclusion was made in a particular study setting. Darwich et al. retrospectively [15] compared the risk of RIFA resistance between patients with prosthetic joint infections treated with regimens involving either immediate or delayed RIFA administration. The first group received additional RIFA only after pathogen detection, while the second group received it directly postoperatively. RIFA resistance increased significantly from 12% in the first group to 19% in the second, whereas the treatment failure rate remained the same (16%) [15]. Of note, in this setting, the patients received RIFA even before knowing whether it was susceptible, and starting from a general RIFA prevalence of 16%, which is very different from Switzerland. 

Many ID physicians and orthopedic surgeons are prone to abandoning this artificial delay and slowly changing their daily practices nowadays [6]. One larger prospective randomized trial investigating (staphylococcal) prosthetic joint infections, the Roadmap Trial^™^, will not delay RIFA therapy https://www.roadmaptrial.com/ (accessed on 1 September 2025). The most recent US recommendations on cardiovascular implantable device infections do not even mention delaying RIFA during bacteremia [28]. The latest French recommendations state that “when surgical treatment is needed, RIFA should be introduced only after the surgical reduction of the bacterial inoculum” but do not mention ideal timing or the presence of discharging wounds [12], for which there is equally very sparse literature. Additionally, basic researchers have no difficulties when testing local RIFA powder, specifically for its anti-biofilm properties, regarding traumatic contaminated wounds [29] and have not found RIFA resistance after screening [29]. Likewise, topical RIFA as monotherapy is equally used for open neural tube defects to prevent ventriculoperitoneal shunt infection without reported resistance problems [30]. Similarly, clinicians may use RIFA with or without antibiotic combinations to eradicate *S. aureus* skin carriage from healthy and sick populations [31]. In an extensive microbiological review of RIFA use in human medicine [3], there is no mention of delaying the prescription because of a high inoculum or ulcerations for orthopedic surgery, nor for ulcerative mycobacteria [32]; additionally, there are no therapeutic recommendations regarding RIFA use for tuberculosis.

In summary, we can reasonably exclude a major concern regarding “early” RIFA introduction, provided that the infective inoculum has been largely reduced by surgical debridement. Generally speaking, a high inoculum does not necessarily lead to resistance of antibiotics nor any other antibiotic agent in daily clinical use but instead leads to failure of (local) infection control. Theoretically, early RIA introduction would enhance infection control, at least for bacteria embedded in the biofilm. The second frequently cited concern is open wounds in the postoperative period. Clinicians who withhold RIFA until advanced wound closure (which might take up to three weeks postoperatively) have no convincing rationale for this practice. In contrast to possible mutations because of a high mass of pathogens, open wounds reveal another pathogenesis of resistance. Current therapeutic antibiotic agents select new strains on the patient’s skin and in the hospital environment that continuously enter the body. This problem does not concern RIFA or staphylococci alone. Indeed, open wounds do not only lead to RIFA resistance among new staphylococcal strains, but they also lead to new selections of other bacteria, such as Gram-negatives. This risk of secondary surgical site infections can reach 10% in orthopedic surgery [33]. Leung et al. investigated the risk of RIFA-resistant staphylococci in open-wound vascular surgery [34]. Unsurprisingly, compared to surgeries with primarily intraoperative closure, surgeries with open wounds show that RIFA-resistant strains are selected significantly more often within only 14 days at maximum, suggesting selection occurs via the open wound and not per mutation. Of note, the authors did not investigate the timing of RIFA introduction nor the selection of other pathogens [34].

In terms of RIFA dosing in infected arthroplasties, two French trials showed that lower doses are as efficient and safe as the recommended high-dose regimen in France that exceeds the internationally accepted amount of 600 mg to 900 mg per day [8,24], and we agree with these findings. 

Our study has limitations: Firstly, it is a retrospective single-center study with an apparently small sample size, which limits the generalizability of the results on a global scale. Even in Switzerland, there are many centers that do not withhold RIFA beyond 1 to 2 days following surgical debridement. However, with a sample size of over a hundred episodes, we are confident that the data are robust and that we can avoid underpowering. Given our large study population, our study represents one of the largest sample sizes in the literature regarding this topic. Secondly, we might not be aware of control samples outside of our hospital, notably from superficial swabs in the outpatient setting or in the general practitioner’s office. Similarly, we ignore whether the patients actually took their RIFA medication as prescribed, especially in the outpatient setting. RIFA is an antibiotic with the most frequent AEs in orthopedic infections (e.g., nausea, emesis, red urine coloration, and/or hepatitis) and regularly interacts with other essential medications [3], including antibiotic agents [12,27,35], and nutritional interventions. The incidence of important AEs (with consequent halting of RIFA therapy or by modifying it) largely varies in the literature, ranging from 10% [16] and 13% [26] to 18% [36], 21% [25], 24% [8], and 71% when we include light events [17]. Unsurprisingly, according to a survey from Germany, the proportion of patients who discontinued RIFA (without replacement) due to attributed adverse events was 19% [37]. In evaluations conducted in France and Switzerland, a full treatment course with RIFA could only be achieved in 75%–82% of cases [8,9]. In light of this, the use of biosensors and analytical methods to optimize the individual RIFA dose is gaining momentum [38]. Thirdly, resistance in our study was based on standard semi-automatized susceptibility testing and not on clinical grounds, the detection of heterogeneity in a research laboratory, or the routine determination of minimal inhibitory concentrations [10,39]. Lastly, we computed the delay in RIFA introduction with the number of objective days that may serve as an arguable surrogate for wound problems, which can only be assessed in prospective trials.

## 4. Materials and Methods

### 4.1. Setting, Study Objective, and Criteria

The Balgrist University Hospital is a tertiary referral orthopedic center in Zurich, Switzerland. It maintains a prospective cohort of all documented infections and has performed multiple retrospective and prospective trials of orthopedic infections since July 2018. This is a study of orthopedic infections with an emphasis on RIFA-related clinical and microbiological variables. We aimed to investigate whether the timing of postoperative RIFA introduction influences overall clinical outcomes, infection recurrences, or RIFA resistance among future staphylococcal species. The inclusion criteria are an orthopedic implant infection due to any staphylococci, the surgical retention or exchange of infected material, a meticulous documentation of antibiotic treatment and AE, and an active follow-up of at least one year. Exclusion criteria are mixed infections with a predominance of non-staphylococcal pathogens, a history of therapy with RIFA or its derivates for any reason, a lack of detailed information, and a lack of a Balgrist-specific signed general consent.

### 4.2. Study Definitions and Microbiological Cultures

We diagnosed a bacterial infection based on clinical, radiological, and microbiological criteria according to international guidance, basically consisting of the presence of local inflammation/pus that is microbiologically confirmed by the presence of the same *staphylococcus* strain in at least two deep intraoperative tissue samples and approved by our infection specialists. Our study had two study endpoints (outcomes): “clinical failure” and “microbiological recurrence” occurring after the end of therapy. We defined “clinical failures” as the need for surgical revision, or any new therapy, in the former infection site for any reason, including for infection relapses. A relapse with the same pathogen (according to its antibiotic susceptibility testing) as in the index infection was labeled as a “microbiological recurrence”. In this definition, “microbiological recurrence” is an integral part of “clinical failures”, but not every “clinical failure” is a “microbiological recurrence”. The reason for having two endpoints is crucial for studies on antibiotic treatments. “Clinical failures” are clinically more important, especially from a surgical point of view. In contrast, we can incriminate antibiotic agents only for the outcome of “microbiological recurrences”. Often, researchers distinguish a third form of failure, which is a “persistence” of infection despite week-long treatment. The outcome of “persistence” is, however, arbitrary to define. In this study, we are only interested in the outcomes after the end of scheduled antibiotic treatment.

The “Institute for Medical Microbiology” at the University of Zurich performed all bacteriological examinations using the EUCAST criteria [39] on standard agars and enrichment broths, with a fixed incubation period of 14 days for implant-related infections. The microbiologists were blinded to this study and determined the Minimal Inhibitory Concentration (MIC) breakpoint automatically. Only in the case of unclear results and/or concomitant independent microbiological studies did they use complementary techniques such as polymerase chain reaction (PCR). Pathologists determined the presence of inflammation (infection) in histology without providing information on the species. We considered two staphylococci strains as identical if they belonged to the same species and revealed the same susceptibility testing results (with slight differences). Conversely, we arbitrarily determined two identical species as being different if they differed in at least three antibiotic classes according to routine susceptibility testing. We renounced typization and Therapeutic Drug Monitoring for antimicrobials, including for RIFA [40].

### 4.3. Statistical Analysis Plan

A prospective randomized trial cannot provide an answer for our study question and would not be ethical. Thus, embedded in a retrospective case–control study design, we linked RIFA-related antibiotic variables to the occurrence of RIFA-resistant strains and to the general therapeutic success of infection. As we expected very few new RIFA-resistant strains and “microbiological recurrences”, the analyses were mainly descriptive. However, the case mix was expected to be large, which we adjusted by performing a multivariate Cox regression analysis with the outcome “clinical failure”, for which the infection episodes were censored at the date of death, occurrence of therapeutic failures, or latest visit to our hospital. The number of variables in the final model was limited to the ratio of 1 variable to 5 to 8 outcome events [41], which also excluded a multivariate model for the potential outcome of “microbiological recurrence” or RIFA resistance due to paucity of events. Practically, we chose the following variables for the final model: delay in RIFA introduction, total duration of RIFA administration, and daily RIFA doses. We computed “delay in RIFA” as a continuous and categorized variable (≤3 days, 4–7 days, and >8 days).

All study patients benefit from long-lasting RIFA combination therapy. In that sense, our final model did not allow to compute for the effectiveness of the presence (or absence) of RIFA use in terms of clinical outcomes [42,43]. We used STATA^™^ software (Version 19; College Station, TX, USA) and considered *p*-values ≤0.05 (two-tailed) as significant.

## 5. Conclusions

In our retrospective cohort of staphylococcal orthopedic implant infections among adult patients, and in line with most studies available in the literature, the timing of postoperative RIFA introduction failed to influence the risk of overall “clinical failures”, the incidence of microbiologically identical infection, or the occurrence of ultimate infection or future colonization with RIFA-resistant staphylococci.

## Figures and Tables

**Table 1:** Univariate and multivariate associations with the outcome “clinical failure”. (Cox regression analyses with results expressed as hazard ratios with 95% confidence intervals).

*n* = 103	Univariate Results	Multivariate Results
Infection due to *Staphylococcus aureus* (vs. coagulase negatives)	0.9 (0.4–2.0)	1.0 (0.4–2.4)
Methicillin-resistant staphylococci	1.8 (0.8–4.2)	-
Delay in rifampicin introduction (continuous variable)	1.2 (0.8–1.1)	1.2 (0.9–1.7)
- Delay rifampicin by ≤ 3 days	1 (default)	1 (default)
- Delay rifampicin by 4–7 days	1.4 (0.5–3.9)	1.4 (0.5–4.2)
- Delay rifampicin by ≥ 8 days	1.7 (0.6–5.1)	2.0 (0.6–6.1)
Total duration of rifampicin prescription (continuous variable)	1.0 (1.0–1.0)	1.0 (1.0–1.0)
Daily rifampicin dose (continuous variable)	1.0 (1.0–1.0)	1.0 (1.0–1.0)
Arthroplasty infections	1.0 (0.4–2.2)	0.9 (0.4–2.2)
Number of past revisions	**9.5 (2.2–40.1)**	-

Statistically significant results are displayed in *bold*. “ - ” = not included in the model due to interaction.

## Data Availability

Anonymized key data may be provided upon reasonable scientific request to the corresponding author.

## Supplementary Materials

The following supporting information can be downloaded at: https://www.mdpi.com/article/10.3390/antibiotics14101043/s1, Supplementary S1 resumes our narrative literature review on our study topic.
