# Supplementary material for: Evaluating the Link Between Postoperative Timing of Rifampicin Introduction and the Clinical and Microbiological Outcomes of Orthopedic Staphylococcal Implant Infections
Source: Antibiotics (Basel). 2025 Oct 17;14(10):1043. doi: 10.3390/antibiotics14101043 (PMC12561295; doi:10.3390/antibiotics14101043)
Supplement: Supplementary file 1 [file antibiotics-14-01043-s001.zip › antibiotics-3896715-supplementary.pdf]

Study Details	Design	Antibiotic regimen	Duration, Timing	Pathogens	Population	Summary
Archer, **1983**, USA [4]	*In vitro* and *in vivo* experimental study	Rifampin in combination with vancomycin, gentamicin, or cephalothin	Rifampin Therapy initiated 1–6 days post-infection.	MRSE	*In vitro* rabbit endocarditis model	Rifampin-based combinations were more effective. Rifampin resistance was prevented when used in combination.
Aydın, **2021**, Turkey [43]	Meta-analysis (Frequentist & Bayesian)	Rifampin combined with various companion antibiotics; regimens varied across studies	Duration and timing varied across the 13 observational studies; rifampin continued for several weeks	MSSA, MRSA and CoNS	PJI	Meta-analysis revealed that rifampin-based combination therapy was associated with higher treatment success, despite heterogeneity and potential bias.
Baumgärtner, **2024**, Germany [42]	Retrospective cohort study	Rifampin-based regimens; dosage not specified	Within first postoperative week; duration 6–12 weeks	*S. aureus,* CoNS	Acute and chronic PJI. Rifampicin-sensitive PJI (group 1): 35 patients. Rifampicin-resistant PJI (group 2): 28.	Rifampin-resistant PJIs were associated with significantly worse functional outcomes, regardless of infection chronicity.
Becker, **2020**, France [24]	Retrospective multicenter study	Rifampin-based regimens combined with other antibiotics.	Rifampin initiated at median 8.5 days after debridement: total duration 85 days.	*S. aureus* (n=65, 82.3%), incl. MRSA (n=7, 10.8%) and CoNS (n=16, 20.3%)	Early-onset acute PJI treated with DAIR	Prolonged rifampin therapy (>14 days) significantly improved treatment success.
Brinkman, **2015**, USA [13]	*In vivo* rat model (foreign-body)	Rifampin monotherapy (25 mg/kg q12h)	Rifampin started after infection established;	MRSA	Rats with foreign-body	Resistance emerged during monotherapy but disappeared after

			continued for 21 days			cessation. Retreatment favored susceptible strains. I
Brinkman, **2017**, USA [7]	In vivo rat model (foreign-body osteomyelitis)	Rifampin monotherapy (25 mg/kg q12h) followed by combined rifampin–vancomycin	Rifampin started after establishing MRSA infection; monotherapy for 21 days, then combo therapy per protocol	MRSA	Rats with foreign-body osteomyelitis model	Initiating rifampin monotherapy, followed by combination therapy, significantly reduced bacterial counts; rifampin resistance emerged, but was mitigated by subsequent combined therapy.
Czekaj, **2011**, Switzerland [16]	Single-center sase series in Geneva<br><br>Investigates clindamycin with rifampicin	Oral clindamycin + rifampin (300 mg q6h + 450 mg q12h)	Started ≤48 h post-debridement; median duration 45 days	12 MSSA, 5 MRSA; 3 CoNS	20 patients with staphylococcal osteoarticular infections	High treatment success (>90%) with oral clindamycin–rifampin regimen; supports feasibility of fully oral treatment in selected cases.
Coiffier, **2013**, France [12]	Review – *in vitro*, animal, clinical	Rifampin 600 mg/day orally in combination with other antibiotics	Rifampin initiated after surgical debridement; exact timing not specified	*Staphylococcus* spp., exact subgroup numbers not reported	Patients and models with staphylococcal bone and joint infections	Highlights the importance of companion antibiotics to prevent rifampin resistance and improve biofilm efficacy.
Darwich, **2021**, Germany [15]	Retrospective cohort study	Rifampin orally 600 mg once daily (with other standard antibiotics)	Group 1 (n=25): Rifampin started after pathogen detection—mean 8.3 ± 2.5 days (range, 4–11 d).	*S. epidermidis*; rifampin resistance in 14/117 (12%) in Group 1, 19%, in Group 2 86%	62 PJI patients (322 surgical episodes) treated with DAIR or similar procedures	Immediate rifampin initiation significantly increased emergence of rifampin resistance (19% vs. 12%, $p < 0.05$), without

			Group 2 (n=37): Rifampin started immediately			affecting treatment failure rates.
Falagas, **2007**, Greece [31]	Systematic review of RCTs and comparative trials	Oral rifampin 600 mg once daily or in combination with systemic/topical agents (e.g., ciprofloxacin, minocycline, fusidic acid, vancomycin)	Regimens began at entry; typical durations (5 days TMP/SMX + rifampin, 14 days rifampin + minocycline)	*S. aureus* (methicillin-susceptible and resistant); total n = 282 patients	Patients with body carriage of *S. aureus*, including MRSA	Rifampin-based regimens significantly improved eradication of *S. aureus* carriage. Resistance development in 0–40 % (overall 17 %).
Ferry, **2010**, Switzerland [10]	Retrospective cohort study	Rifampin (mostly 600 mg once daily) in combination with fusidic acid or other antimicrobials	Rifampin initiated post-debridement; exact day not specified	Only MRSA: 52 patients; 41 isolates tested, all vancomycin-susceptible (MIC ≤ 2 mg/L)	Patients with MRSA orthopedic device-related infections (n = 52)	Rifampin-free therapy independently predicted treatment failure. Combination of rifampin + fusidic acid 78% cure vs. 17% with mono-therapy in DAIR.
Garzoni, **2014**, Switzerland [33]	Prospective pilot study. Efficacy and interaction between rifampicin and flucloxacillin *in vivo*	Continuous IV flucloxacillin 12 g/day + oral rifampin 600 mg bid	Rifampin added after culture confirmation (~72 h after flucloxacillin)	*S. aureus* (n=15; all cases mono-infection)	15 adult patients with bone/soft-tissue MSSA infections	Rifampin increased flucloxacillin serum levels by 44.5% in 10/15 patients; all infections cured.
Grünwald, **2025**, Germany [6]	Retrospective cohort study	Rifampin 600 mg/day (oral/intravenous) combined with other antibiotics	Rifampin initiated on postoperative Day 2. long-term per protocol (~6–12 weeks)	*S. aureus* and CoNS	212 with revision for PJI (hip or knee)	Low rifampin resistance rates: 0.9% at spacer explantation, 1.4% at follow-up (55 months). Early initiation on Day 2 was safe.
Karlsen, **2020**, Norway [26]	Multicenter randomized controlled trial	Rifampin 300 mg × 3 p.o. + Cloxacillin (2 g × 4 IV → 1 g × 4 oral) for MSSA/CoNS;	Rifampin started Day 1 post-surgery; rifampin + cloxacillin	MSSA: 34 isolates; MRSE: 10 isolates; also *S.*	Acute PJIs with DAIR (n = 48)	No significant difference in 2-year remission: rifampin

		Rifampin + Vancomycin (1 g × 2 IV) for MRSE	continued 6 weeks (2 weeks IV, 4 weeks oral);	*lugdunensis, S. capitis* (1 each)		group 74% vs. monotherapy 72%. No rifampin resistance detected among failures.
Kobayashi, **2024**, Japan [22]	Systematic review & meta-analysis (27 studies)	Rifampin combined with various agents; common doses: 600 mg once daily	Rifampin initiation after surgery, exact timing variable between studies; for 4–12 weeks	*Staphylococcus* spp., including *S. aureus*, CoNS, Cutibacterium	Orthopedic implant–related infections	Rifampin significantly improved cure rates in cohort studies, especially with fluoroquinolones; not confirmed in RCTs.
Kruse, **2022**, Canada [25]	Systematic review & meta-analysis (33 studies)	Rifampin combined with standard regimens; common doses: 600 mg daily and orally	Rifampin started postoperatively, exact day varied; continued typically 4–12 weeks	*S. aureus* and CoNS	PJI treatment	Rifampin reduced treatment failures (26% vs. 36%) in exchange arthroplasty, not DAIR. Rifampicin adverse events 21%.
Kugelman, **2025**, USA [37]	Retrospective cohort study	Rifampin (dosage per protocol, e.g., 600 mg daily)	Rifampin in 49%; start timing not specified; 19% discontinued before the end of 6-week course	S. aureus PJIs (DAIR or one-stage revision)	PJI patients (n = 87)	19% of patients discontinued before 6 weeks. Main barriers: drug–drug interactions, GI issues, and AKI.
Lora-Tamayo, **2013**, Spain [21]	Prospectiv nationwide multicenter observational cohort	Rifampin 600 mg/day p.o. combined with levofloxacin 500 mg/day p.o. (or cloxacillin/linezolid for MSSA/CoNS)	Rifampin started within 48 h post-DAIR; median duration 12 weeks (IQR 8–16 weeks)	MSSA: n = 152; MRSA: n = 24; CoNS: n = 64 (total cohort n = 240)	Acute *S. aureus* and CoNS PJI treated with implant retention	2-year treatment success: 83% in MSSA, 75% in MRSA, 68% in CoNS. Early rifampin initiation associated with better outcomes.
Perlroth, **2008**, USA [20]	Systematic review (*in vitro*, animal, clinical)	Rifampin 300–1,200 mg/day (oral or IV) combined with antibiotics such as	Rifampin initiated at study entry (timing varies by trial)	MSSA and MRSA	Patients and animals with implant-related	Animal studies show benefit of adjunctive rifampin in bone and device infections. In

		vancomycin, ciprofloxacin, oxacillin, nafcillin			*S. aureus* infections	human trials, the combined therapy shows promise, but lacks evidence.
Petersen, **2025**, Denmark [5]	*In vitro* laboratory model for rifampicin resistance in *S. aureus*	Rifampicin alone or in combination with levofloxacin, clindamycin, fusidic acid (lab concentrations, not clinical dosing)	Long-term serial passaging of S. aureus under antibiotic exposure	*S. aureus*	Laboratory model of S. *aureus* evolution under antibiotic pressure	Rifampicin mono-therapy selected resistance; combination (fusidic acid or levofloxacin) delayed resistance.
Pupaibool, **2024**, USA [3]	Narrative review of clinical and experimental evidence	Rifampin standard doses (~600 mg daily orally or IV) combined with β-lactams, fluoroquinolones	Rifampin typically initiated after surgery within 2–7 days, duration 6–12 weeks	Biofilm-associated, *S. aureus* and CoNS	PJIs	Highlights rifampin's biofilm penetration, synergy, and improved outcomes in PJI. Notes few RCTs.
Yusuf, **2024**, Netherlands [23]	Systematic review and meta-analysis (27 studies, >3,500 patients)	Rifampicin 600–900 mg/day orally, combined with fluoroquinolones or beta-lactams	Rifampicin started within 5–7 days post-surgery; duration 2–12 weeks	*S. aureus,* CoNS, *Streptococcus*	Implant-associated infections (PJI, fracture fixation, vascular grafts)	Rifampicin improved outcomes in *Staphylococcus* infections (OR 2.1 for *S. aureus*); no benefit for streptococci
Reinecke, **2023**, Germany [17]	Prospective cohort study	Oral rifampin (dose per protocol not specified) + co-trimoxazole (dose not specified)	Oral therapy including rifampin began after initial 14-days of IV antibiotics	Not pathogen-specific (PJI etiologies varied)	80 PJI patients undergoing single-/two-stage exchange or DAIR	73 patients (91%) had rifampicin-related adverse events; 46% gastrointestinal (nausea, diarrhea) and 22% on the skin. Antibiotic stopped in 6%, and dose adjustments in 29%.
Schindler, **2013**, Switzerland [36]	Retrospective cohort study	Oral rifampin 600 mg/day (in combination with various long-term antibiotics)	Rifampin started during long-term therapy (~week 3 median, after ~21 days of antibiotic	Not pathogen-specific; osteoarticular infections (n = 393 episodes);	Patients with osteoarticular infections on long-term	29% had adverse events prolonging hospital stay; rifampin use was protective against *C. difficile*

			therapy including 2 weeks IV); total course 8 weeks	*Clostridioides difficile* in 14 (3.6%) cases	antibiotic regimens	colitis (HR 0.2, 95%CI 0.05–0.97).
Shiels, **2018**, USA [29]	*In vivo* rat post-traumatic infection prophylaxis model	Topical rifampin powder 50 mg – applied directly to femoral defect (alone or combined with vancomycin, daptomycin powder)	Applied immediately into the contaminated defect during surgery (Day 0)	*S. aureus* (animal-inoculated)	Rats with delayed musculoskeletal trauma infection	Topical rifampin powder significantly reduces bacterial counts and infection signs compared to vancomycin or daptomycin. No rifampin resistance.
Tonnelier, **2021**, France [8]	Retrospective single-center cohort study	Rifampin success categorized by its dose	Rifampin started 5–7 days post-surgery (after IV antibiotics); for 6–12 weeks	Rifampin-sensitive *Staphylococcus* (411 PJIs)	PJI due to staphylococci	Cure rates similar across dosing groups: 67% (<10 mg/kg), 76% (10–20 mg/kg), 69% (>20 mg/kg).
Widmer, **1992**, Switzerland [9]	Prospective clinical study	Rifampin 600 mg/day p.o. (or IV) combined with a β-lactams or ciprofloxacin 500 mg bid	Rifampin started after diagnosis; duration 86 days	*Staphylococcus* spp.	11 retained implant-related infections	82 % treatment success.
Zimmerli, **1998**, Switzerland [18]	Randomized, double-blind RCT (landmark study)	Rifampin 600 mg/day (oral) + ciprofloxacin 500 mg twice daily, after 2 weeks of IV flucloxacillin or vancomycin	Rifampin initiated after 2-week IV therapy, at start of oral phase; continued during 3–6 months	MSSA (n = 26) and *S. epidermidis* (n = 7)	33 patients with orthopedic staphylococcal implant-related infections. Early termination due to high efficacy of combination	Cure was 100% (12/12) in rifampin group vs. 58% (7/12) in placebo group (p = .02); no rifampin resistance emerged.

MRSA - Methicillin-resistant *Staphylococcus aureus;* MSSA - Methicillin-sensitive *Staphylococcus aureus*
PJI - Prosthetic joint infections; DAIR- Debridement, Antibiotics, and Implant Retention
CoNS - Coagulase-negative staphylococci; TMP – Trimethoprime; SMX - Sulfamethoxazole
IV- intravenous; AKI - acute kidney impairment; CI – Confidence Interval
